# The role of incoming flow on crystallization of undercooled liquids with a two-phase layer

**DOI:** 10.1038/s41598-022-22786-w

**Published:** 2022-10-25

**Authors:** Dmitri V. Alexandrov, Liubov V. Toropova

**Affiliations:** 1 Laboratory of Multi-Scale Mathematical Modeling,Department of Theoretical and Mathematical Physics, Ural Federal University, Lenin Ave., 51, Ekaterinburg, Russian Federation 620000; 2 Otto-Schott-Institut für Materialforschung, Friedrich-Schiller-Universität-Jena, 07743 Jena, Germany; 3 Laboratory of Mathematical Modeling of Physical and Chemical Processes in Multiphase Media,Department of Theoretical and Mathematical Physics, Ural Federal University, Lenin Ave., 51, Ekaterinburg, Russian Federation 620000

**Keywords:** Applied mathematics, Phase transitions and critical phenomena

## Abstract

Motivated by important applications of crystallization phenomena, we consider a directional solidification process for a binary melt with a two-phase (mushy) layer in the presence of weak melt flow. We consider the steady-state solidification scenario, so that the two-phase layer filled with solid and liquid material keeps its thickness. In addition, we consider that the melt flows onto the two-phase layer slowly in the opposite direction to directional crystallization and solidifies there. A complete analytical solution to non-linear two-phase layer equations is constructed in a parametric form, where the solid phase fraction represents a decision variable. The temperature and solute concentration distributions, mushy layer permeability and average interdendritic spacing as well as solidification velocity and mushy layer thickness are analytically determined. We show that incoming melt flow plays a decisive role on mushy layer parameters and internal structures. The solid phase fraction within the two-phase layer and its thickness essentially grow while the mushy layer permeability and average interdendritic spacing decrease with increasing intensity of incoming melt flow.

## Introduction

It is well known that a flat interface between the solid and liquid phases in the crystallisation processes of undercooled melts and supersaturated solutions can be morphologically unstable. The physical cause of such instability is thermal/concentration undercooling, anisotropy, fluid currents, melt convection as well as fluctuations of external parameters governing the crystallization process (e.g. atmospheric temperature or under-ice friction velocity)^1–11^. The evolution of morphological instability leads to the growth of patterns and dendrite-like structures ahead of the crystallisation front. These growth formations form a two-phase (mushy) layer ahead of the front, filled with solid and liquid phases. In other words, the phase transformation from the undercooled liquid state takes place within this two-phase layer, which moves towards the melt due to the cooling of the solid material. Note that this layer completely changes the crystallization scenario. So, for example, the temperature at each point of this layer is lower than the crystallisation temperature and the growing patterns and dendrites release the latent heat of phase transformation and thus partially compensate for the undercooling. In addition, the growing solid phase displaces the dissolved impurity in front of it which lowers the crystallisation temperature according to the phase diagram. These processes lead to the formation of complex branching structures of the solid phase, the gaps between which are filled by a liquid with a higher impurity concentration. Gravity is usually present in experimental facilities and natural processes and can be the cause of natural convection^12–14^. In addition, fluid currents in electromagnetic levitation apparatuses and natural processes can also lead to convection (see, among others,^15,16^). Convection is therefore one of the most important factors affecting the structure of the two-phase layer and the crystallisation process as a whole. Since the equations for convective heat and mass transfer are considerably more complex than similar equations in its absence, convective flows are usually analysed numerically^17–19^.

In this study, we develop the analytical theory of a weakly flowing liquid (melt) into a two-phase region where freezing (solidification) of this liquid occurs. This approximation allows us to construct an analytical solution to the nonlinear problem with moving boundaries taking into account (i) the quasi-equilibrium structure of the two-phase layer (when undercooling is fully compensated by the latent heat of crystallization) and (ii) a constant crystallization rate. For solving the problem with two moving boundaries, we used the method of transition to a new independent variable, the solid phase fraction, which was previously developed in Refs.^20,21^. The resulting solution establishes the effect of the fluid flow rate on the two-phase region and the characteristics of the solid phase. This article is organised as follows. The model of convective heat and mass transfer in all the phases is formulated in Section "The model". Its complete analytical solution is constructed in Section "Analytical solutions". Behaviour of these solutions is discussed in Section "Behaviour of solutions". The main outcomes of the present theory are summarized in Section “Conclusion”.

## The model

**Figure 1:**
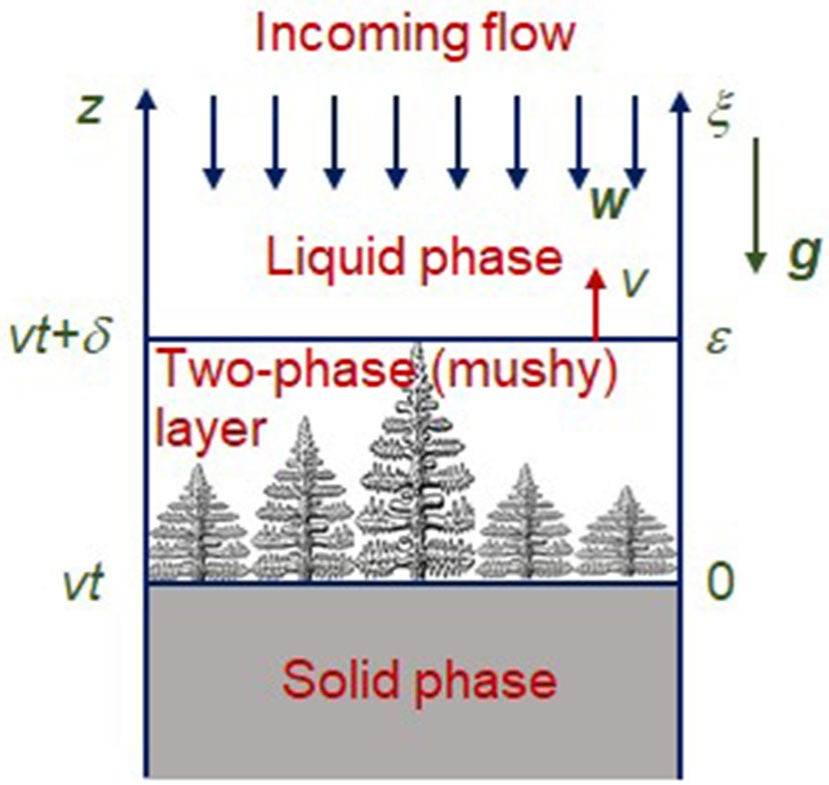
A scheme of directional steady-state crystallization with a mushy layer in the presence of incoming flow. The origin of moving coordinate system is at the boundary solid phase—two-phase layer.

Consider a directional crystallisation process with constant velocity *v* along the spatial coordinate *z*, schematically illustrated in Fig. 1. Here the spatial axis *z* corresponds to the laboratory coordinate system while axis $\xi$ moves together with a mushy layer ($\xi =v\left( z-vt\right) /D_l$, *t* is time and $D_l$ is the diffusion coefficient of solute). The two-phase layer of length $\delta = \varepsilon v/D_l$ lies between purely solid ($\xi <0$) and liquid ($\xi >\varepsilon$) phases ($\varepsilon$ is the dimensionless two-phase layer thickness). As this takes place, there is a weak flow of undercooled liquid in the opposite direction. For simplicity, we consider the case when this liquid is completely frozen in the two-phase layer.

The convective heat and mass transfer equations are as follows

1 $$\begin{aligned} \rho (\psi )c(\psi )\frac{\partial T}{\partial t}+\rho _lc_l\mathbf{W}\cdot \nabla T= & {} \nabla \cdot (k(\psi )\nabla T) +Q_V\frac{\partial \psi }{\partial t}, \end{aligned}$$

2 $$\begin{aligned} \frac{\partial }{\partial t}\left( (1-\psi )C \right) +\mathbf{W}\cdot \nabla C= & {} \nabla \cdot (D(\psi )\nabla C) -k_eC\frac{\partial \psi }{\partial t}, \end{aligned}$$

where *T* and *C* stand for the temperature and solute concentration, $\psi$ is the solid phase fraction ($\psi =1$ in solid, $0\le \psi \le 1$ in the two-phase layer, and $\psi =0$ in liquid), $Q_V$ is the latent heat parameter, $k_e$ is the equilibrium partition coefficient, $\mathbf{W}$ is the volume flow of interdendritic liquid so that $\mathbf{W} = (1-\psi )\mathbf{w}$^7,22,23^ (barring dendrite erosion and breakage in the two-phase layer), $\mathbf{w}$ is the local velocity of liquid. Note that $\mathbf{W}$ satisfies the continuity law $\nabla \cdot \mathbf{W} =0$. The density $\rho$, specific heat *c*, thermal conductivity *k* and diffusion coefficient *D* are dependent of $\psi$ and defined, for simplicity, through the following linear functions^24^

3 $$\begin{aligned} \rho (\psi )c(\psi ) = \rho _sc_s\psi +\rho _lc_l(1-\psi ),\ k(\psi ) = k_s\psi +k_l (1-\psi ),\ D(\psi ) =D_l(1-\psi ), \end{aligned}$$

where subscripts *s* and *l* designate the solid and liquid phases, respectively. Here we traditionally neglect diffusion in solid. Note that these expressions give exact results for a laminated medium when there is no component of the heat flux normal to the planes of the laminates. Since it is found experimentally that the primary dendrites are aligned with the mean thermal gradient, these expressions are likely to give reasonable approximations for the description of a two-phase layer. Also, we describe here vertically oriented dendrites in the mushy layer found experimentally and simulated numerically^25,26^.

Considering the model of quasiequilibrium two-phase layer, we relate temperature and solute concentration from the phase diagram as

4 $$\begin{aligned} T=T_* - f(C), \end{aligned}$$

where $T_*$ is the phase transition temperature for the pure system (for $C=0$) and *f*(*C*) is the concentration-dependent function. For example, dealind with the linear phase diagram, we have $T=T_*-m_eC$, where $f(C)=m_eC$, and $m_e$ is the equilibrium slope of liquidus line. If the liquidus equation deviates slightly from the linear relationship, a quadratic function should be used^27^. In the more general case, Eq. (4) defines the experimentally known relationship between crystallisation temperature and solute concentration.

The volume flux $\mathbf{W}$ of interdendritic liquid is connected with the permeability $\Pi (\psi )$ of two-phase layer and pressure *p* by means of Darcy’s law^7,23,28^

5 $$\begin{aligned} \frac{\eta \mathbf{W}}{\Pi (\psi )} = (\rho _l-\rho _c)\mathbf{g} -\nabla p. \end{aligned}$$

Here $\mathbf{g}$ is the gravitational acceleration, $\eta$ is the dynamic viscosity and $\rho _c$ is a characteristic density of liquid.

An important feature of the problem at hand is the fact that variations in the temperature field lead to variations in the liquid density, which is responsible for natural convection^29^. To account for this important effect, we will use a linear relationship between liquid density and temperature

6 $$\begin{aligned} \rho _l-\rho _c =\rho _c\left[ b_0C-a_0(T-T_*)\right] =\rho _c \left[ b_0C +a_0 f(C) \right] , \end{aligned}$$

where Eq. (4) was taken into account. In the case of linear liquidus line, we get $\rho _l-\rho _c = \rho _c b C$, where $b=b_0+a_0m_e$.

The model Eqs. (1)–(6) should be supplemented with boundary conditions at the phase transition interfaces $\xi =0$ and $\xi =\varepsilon$ (see Fig. 1)

7 $$\begin{aligned} Q_V[\psi ]v =\left[ k\mathbf{n}\cdot \nabla T \right] ,\ \ (1-k_e)C[\psi ] v =\left[ D\mathbf{n}\cdot \nabla C \right] . \end{aligned}$$

Here $[\cdot ]$ indicates a jump in a physical value when crossing the boundary, and $\mathbf{n}$ is the normal vector. It is significant to note that $\psi =\psi _*$ at the solid phase – two-phase layer boundary and $\psi =0$ at the two-phase layer – liquid phase boundary ($\psi _*$ should be found through solving the problem).

## Analytical solutions

So, we consider the steady-state solidification process with established velocity *v* and two-phase layer thickness $\delta$ (Fig. 1). As this takes place, an incoming flow of undercooled liquid freezes in the phase interface $\xi =0$ as well as a solid phase matrix within the mushy layer $0<\xi < \varepsilon$. Let us designate the velocity of this flow at $\xi =0$ through $w_l$. In this case $W=w_l$ at $\psi =0$ (at the two-phase layer - liquid phase boundary).

Using the dimensionless variable $\xi = v(z-vt)/D_l$ and keeping in mind that $\partial /\partial z =(v/D_l)\partial /\partial \xi$ and $\partial /\partial t =-(v^2/D_l)\partial /\partial \xi$, we rewrite the model (1)–(6) in the form

8 $$\begin{aligned} -\frac{d}{d\xi }\left( H(\psi )\Theta \right) +j\frac{d\Theta }{d\xi }=n_1\frac{d}{d\xi }\left[ \kappa (\psi )\frac{d\Theta }{d\xi } \right] -\left[ n_2 +\left( \frac{\rho _sc_s}{\rho _lc_l} -1 \right) \Theta \right] \frac{d\psi }{d\xi }, \end{aligned}$$

9 $$\begin{aligned} -\frac{d}{d\xi }\left[ (1-\psi )\Sigma \right] +j\frac{d\Sigma }{d\xi }=\frac{d}{d\xi }\left[ (1-\psi )\frac{d\Sigma }{d\xi } \right] +k_e\Sigma \frac{d\psi }{d\xi }, \end{aligned}$$

10 $$\begin{aligned} \Theta = \Theta _* -\frac{f(C_\infty \Sigma )}{m_eC_\infty }, \end{aligned}$$

11 $$\begin{aligned} j=-\mathrm{Ra}\ \frac{\Pi (\psi )}{\Pi _0}\left[ \frac{dp_1}{d\xi }+\frac{f_1(\Sigma )}{b}\Sigma \right] ,\ \ f_1(\Sigma ) = b_0+\frac{a_0f(C_\infty \Sigma )}{C_\infty \Sigma }, \end{aligned}$$

where $C_\infty$ is a constant solute concentration in liquid far from the two-phase layer (at $\xi \rightarrow \infty$), and $\Pi _0$ is a reference value of mushy layer permeability $\Pi$. The following dimensionless functions and parameters were used in deriving Eqs. (8)–(11)

12 $$\begin{aligned} \begin{aligned} \Theta =\frac{T}{m_eC_\infty },\ \ \Sigma =\frac{C}{C_\infty },\ \ j=\frac{W}{v}<0,\ \ H(\psi ) = \frac{\rho (\psi )c(\psi )}{\rho _lc_l},\ \ \kappa (\psi ) = \frac{k(\psi )}{k_s},\\ \Theta _* =\frac{T_*}{m_eC_\infty },\ \ n_1=\frac{k_s}{D_l\rho _lc_l},\ \ n_2=\frac{Q_V}{\rho _lc_lm_eC_\infty },\ \ \mathrm{Ra}=\frac{\rho _cbC_\infty g\Pi _0}{\eta v},\ \ p_1 = \frac{vp}{\rho _cbC_\infty gD_l}. \end{aligned} \end{aligned}$$

Here $\Theta$, $\Sigma$ and $p_1$ mean the dimensionless temperature, solute concentration and pressure, $\mathrm{Ra}$ stands for the Rayleigh number, and *j* characterizes the influence of incoming flow. Note that $f_1(\Sigma ) =b$ and $j=-\mathrm{Ra}\ (\Pi /\Pi _0) (dp_1/d\xi +\Sigma )$ in the case of linear liquidus slope. Let us especially emphasize that Eqs. (8) and (9) are dimensionless heat and mass transfer equations in the two-phase layer, while Eqs. (10) and (11) are dimensionless liquidus and Darcy’s equations.

The boundary conditions (7) at mushy layer interfaces read as

13 $$\begin{aligned} \begin{aligned} \frac{n_2}{n_1}(1-\psi _*) =G_s+\kappa (\psi _*)\frac{df/d\Sigma }{m_eC_\infty }\frac{d\Sigma }{d\xi },\ \ (1-k_e)\Sigma +\frac{d\Sigma }{d\xi }=0,\ \ \xi =0, \end{aligned} \end{aligned}$$

14 $$\begin{aligned} \begin{aligned} \frac{d\Theta }{d\xi }=\frac{d\Theta _l}{d\xi },\ \ \frac{d\Sigma }{d\xi }=\frac{d\Sigma _l}{d\xi },\ \ \xi =\varepsilon =\frac{\delta v}{D_l} , \end{aligned} \end{aligned}$$

where $\Theta _l = T_l/(m_eC_\infty )$ and $\Sigma _l =C_l/C_\infty$ designate dimensionless temperature and solute concentration in liquid ($T_l$ and $C_l$ represent their dimensional analogs), $G_s=D_lg_s/(m_eC_\infty v)$, and $g_s$ represents the constant temperature gradient in solid. It means that the temperature $\Theta _s$ in the solid material at a certain distance $\xi =-\xi _o$ from the two-phase layer is known, i.e. $\Theta _s = \Theta _{so}$ at $\xi =-\xi _o$. Note that this condition ensures the steady-state crystallisation velocity *v*.

It is significant to note that

$$\begin{aligned} n_2\gg \left( \frac{\rho _sc_s}{\rho _lc_l} -1 \right) \Theta \end{aligned}$$

for dilute binary undercooled melts^21^. Taking this into account we integrate Eq. (8) with allowance for Eq. (10) and arrive at

15 $$\begin{aligned} \begin{aligned} \frac{d\Sigma }{d\xi }=-\frac{ \left( j-H(\psi ) \right) \left[ \Theta _*- F(\Sigma )\right] +n_2\psi +A}{ n_1\kappa (\psi )dF/d\Sigma },\ \ F(\Sigma )=\frac{f(C_\infty \Sigma )}{m_eC_\infty }, \end{aligned} \end{aligned}$$

where *A* is constant.

Now substituting $d\Sigma /d\xi$ from (15) into the mass balance equation (9) and multiplying the result by $d\xi /d\psi$, we get

16 $$\begin{aligned} \begin{aligned} R_1 (\Sigma ,\psi )\frac{d\Sigma }{d\psi } +R_2 (\Sigma ,\psi ) =0,\ \ \Sigma = \Sigma _\varepsilon ,\ \ \psi =0, \end{aligned} \end{aligned}$$

where $\Sigma _\varepsilon$ is the solute concentration at the boundary between the two-phase layer and liquid, and

$$\begin{aligned} R_1 = \psi -1+j+\frac{F^{\prime \prime }(\Sigma )(1-m_2(\psi )F(\Sigma ))+m_2(\psi )F^{\prime 2}(\Sigma ) }{F^{\prime 2}(\Sigma )},\ \ R_2 = (1-k_e)\Sigma +\frac{m_2^\prime (\psi )F(\Sigma ) -m_1^\prime (\psi )}{F^\prime (\Sigma )}, \\ m_1(\psi ) =-(1-\psi ) \frac{(j-H(\psi )) \Theta _* +n_2\psi +A}{n_1\kappa (\psi )},\ \ m_2(\psi )= -\frac{(1-\psi )(j-H(\psi ))}{n_1\kappa (\psi )}. \end{aligned}$$

Note that Eq. (16) represents the one-point Cauchy problem defining the solute concentration $\Sigma$ as a function of solid phase fraction $\psi$ in the case of arbitrary phase diagram (4) (arbitrary function *f*(*C*)).

Since the linear phase diagram is a very common case, we consider it below, which allows us to simplify the analytical solution considerably. So, we have $f =m_eC_\infty \Sigma$, $F=\Sigma$, $F^\prime = 1$, and Eq. (16) becomes

17 $$\begin{aligned} \begin{aligned} \left( \psi -1 +j+m_2(\psi ) \right) \frac{d\Sigma }{d\psi } + \left( 1-k_e +\frac{dm_2}{d\psi } \right) \Sigma -\frac{dm_1}{d\psi } =0. \end{aligned} \end{aligned}$$

Integrating this equation, we come to the solute concentration $\Sigma (\psi )$ in the two-phase layer (at $0\le \xi \le \varepsilon$):

18 $$\begin{aligned} \begin{aligned} \Sigma (\psi ) = \gamma ^{-1}(\psi )\left( \Sigma _\varepsilon +\int \limits _0^\psi \frac{\gamma (\phi ) (dm_1/d\phi )}{j-1 +\phi +m_2(\phi )}d\phi \right) ,\ \ \gamma (\psi ) = \exp \left( \int \limits _0^\psi \frac{1-k_e+dm_2/d\varphi }{j-1+\varphi +m_2(\varphi )}d\varphi \right) . \end{aligned} \end{aligned}$$

Expression (18) defines the solute concentration in the two-phase layer $0\le \xi \le v\delta /D_l$ (or $0\le \psi <\psi _*$). Now substituting (18) into (4), we obtain the temperature profile in this layer.

A constant temperature gradient $d\Theta _s /d\xi =G_s$ in solid leads to the linear temperature profile in solidified material

19 $$\begin{aligned} \begin{aligned} \Theta _s = \Theta _{so} + G_s (\xi + \xi _o),\ \ -\xi _o< \xi <0 , \end{aligned} \end{aligned}$$

where $\Theta _s =T_s/(m_eC_\infty )$, and $\Theta _s$ and $T_s$ represent dimensionless and dimensional temperatures in solid.

Equations governing temperature $T_l$ and solute concentration $C_l$ in liquid follow from Eqs. (1) and (2) at $\psi =0$ and have the form

20 $$\begin{aligned} \begin{aligned} (j_l-1)\frac{d\Theta _l}{d\xi } =\mathrm{Le}\frac{d^2\Theta _l}{d\xi ^2},\ \ (j_l-1)\frac{d\Sigma _l}{d\xi } = \frac{d^2\Sigma _l}{d\xi ^2}, \end{aligned} \end{aligned}$$

where $j_l = W_l/v$ is the ratio of fluid velocity $W_l$ in the liquid phase and the solidification velocity *v*, and $\mathrm{Le }= k_l/ (D_l\rho _l c_l)$ is the Lewis number.

The heat and mass balances at the two-phase layer – liquid boundary $\xi =\delta v/D_l$ (or $\psi =0$) follow from conditions (14), Eq. (10) written out for linear liquidus $\Theta = \Theta _* -\Sigma$, concentration derivative (15) at $\psi =0$, and $j=j_l$ at $\xi =\delta v/D_l$:

21 $$\begin{aligned} \begin{aligned} \frac{d\Theta _l}{d\xi }=\frac{d\Theta }{d\xi }=-\frac{d\Sigma }{d\xi }=-\frac{d\Sigma _l}{d\xi } =\frac{(j-1)(\Theta _*-\Sigma _\varepsilon )+A}{\mathrm{Le}},\ \ \xi =\delta v/D_l. \end{aligned} \end{aligned}$$

The dimensionless solute concentration far from the two-phase layer is also known, i.e. $\Sigma _l\rightarrow 1$ at $\xi \rightarrow \infty$.

Integrating Eq. (20) and taking the boundary conditions (21) into account, we obtain the temperature ($\Theta _l (\xi )$) and solute concentration ($\Sigma _l (\xi )$) in liquid as well as the boundary concentration ($\Sigma _\varepsilon$):

22 $$\begin{aligned} \begin{aligned} \Theta _l (\xi )=\Theta _*-\Sigma _\varepsilon +\frac{(j_l-1)(\Theta _*-\Sigma _\varepsilon )+A}{j_l-1}\left[ \exp \left( \frac{j_l-1}{\mathrm{Le}}\left( \xi -\frac{\delta v}{D_l} \right) \right) -1 \right] ,\ \ \xi >\varepsilon =\frac{\delta v}{D_l}, \end{aligned} \end{aligned}$$

23 $$\begin{aligned} \begin{aligned} \Sigma _l (\xi )= 1+(\Sigma _\varepsilon -1)\exp \left[ (j_l-1)\left( \xi - \frac{\delta v}{D_l}\right) \right] ,\ \ \xi >\varepsilon =\frac{\delta v}{D_l}, \end{aligned} \end{aligned}$$

24 $$\begin{aligned} \begin{aligned} \Sigma _\varepsilon = \frac{(j-1)(\mathrm{Le}-\Theta _*)-A}{(j-1)(\mathrm{Le}-1)}. \end{aligned} \end{aligned}$$

Now equating temperatures (10) and (19) at the two-phase layer – solid phase boundary $\xi =0$ ($\psi =\psi _*$), we get a transcendental equation for the solid phase fraction $\psi _*$

25 $$\begin{aligned} \begin{aligned} \Sigma (\psi _*)=\Theta _* -\Theta _{so}-G_s\xi _o. \end{aligned} \end{aligned}$$

Here $\Sigma (\psi _*)$ is given by expression (18). Next substituting $d\Sigma /d\xi$ at $\xi =0$ ($\psi =\psi _*$) from (15) into the second condition (13), we find the constant *A*:

26 $$\begin{aligned} \begin{aligned} A = (1-k_e)n_1\kappa (\psi _*) \left[ \Theta _* -\Theta _{so}- G_s\xi _o\right] -\left( j-H(\psi _*) \right) \left( \Theta _{so}+G_s\xi _o \right) -n_2\psi _* . \end{aligned} \end{aligned}$$

Eliminating $d\Sigma /d\xi$ from the boundary conditions (13), we obtain the velocity *v* of crystallization

27 $$\begin{aligned} \begin{aligned} v=\frac{k_sg_s}{(1-\psi _*)Q_V +m_eC_\infty k(\psi _*)(1-k_e)\left[ \Theta _* - \Theta _{so}- G_s \xi _o \right] /D_l}. \end{aligned} \end{aligned}$$

Expression (18) shows that the solute concentration is dependent of variable $\psi$ only. Its derivative $d\Sigma /d\xi$ also depends only on $\psi$ as is seen from Eq. (15). Thus, $d\Sigma /d\xi = (d\Sigma /d\psi )(d\psi /d\xi )$, or $y_1(\psi ) =y_2(\psi ) (d\psi /d\xi )$, where $y_1(\psi ) =d\Sigma /d\xi$ and $y_2(\psi ) = d\Sigma /d\psi$. Taking this into account, we obtain the solid-phase fraction $\psi (\xi )$ in the form of its inverse function $\xi (\psi )$ as

28 $$\begin{aligned} \begin{aligned} \xi (\psi ) = \int \limits _{\psi _*}^{\psi } \frac{y_2(\phi )}{y_1(\phi )}d\phi . \end{aligned} \end{aligned}$$

Here $y_1(\psi )$ and $y_2(\psi )$ should be substituted from expressions (15) and (18), respectively.

The mushy layer thickness $\delta$ follows from (28) with allowance for the boundary condition $\xi =\varepsilon =\delta v/D_l$ at $\psi =0$:

29 $$\begin{aligned} \begin{aligned} \delta = \frac{D_l}{v}\int \limits _{\psi _*}^{0} \frac{y_2(\phi )}{y1(\phi )}d\phi . \end{aligned} \end{aligned}$$

Thus, expressions (15), (18), (19) and (22)–(29) determine the analytical solution of mushy layer model in the presence of incoming flow.

The analytical solution obtained enables us to describe some parameters of the mushy layer internal structure. One of them is the two-phase layer permeability, which depends on the evolution of dendrites and the phase composition of solidified materials. The two-phase layer permeability $\Pi$ at all points where the solid phase grow is determined by the solid phase fraction $\psi$. Following the works^17,18,30^, we use here the following dependence

30 $$\begin{aligned} \begin{aligned} \Pi (\psi ) =\Pi _0(1-\psi )^3, \end{aligned} \end{aligned}$$

where $\Pi _0$ is a reference value of permeability. Now combining expressions (28) and (30), we obtain the permeability as an inverse function of spatial coordinate $\xi$ in the two-phase layer

31 $$\begin{aligned} \begin{aligned} \xi (\Pi ) = \int \limits _{\psi _*}^{1-\left( \Pi /\Pi _0\right) ^{1/3}} \frac{y_2(\phi )}{y_1(\phi )}d\phi . \end{aligned} \end{aligned}$$

Another important parameter characterizing the two-phase layer is the average interdendritic spacing $\lambda _1$, which reads as^31^

32 $$\begin{aligned} \begin{aligned} \lambda _1 =\sqrt{\frac{2\pi \rho }{d_a \vert \partial \psi /\partial z \vert _{z=vt}}}= \sqrt{\frac{2\pi \rho _{dt} D_l }{d_a v }\left| \frac{y_2(\psi _*)}{y_1(\psi _*)} \right| }, \end{aligned} \end{aligned}$$

where $\rho _{dt}$ is the dendrite tip diameter, and $d_a=1$ and $d_a=0.86$ for cubic and hexagonal dendritic arrays. To find $\rho _{dt}$ we use the selection theory of stable dendritic growth^16,32^, which leads to

33 $$\begin{aligned} \begin{aligned} \rho _{dt} = \sqrt{\frac{2d_0a_l}{v\sigma _0\beta ^{7/4}P}},\ \ P= 1+\frac{2m_eC_\infty \Sigma _{ds}(1-k_e)k_l}{Q_VD_l}, \end{aligned} \end{aligned}$$

where $d_0$ is the capillary constant, $a_l=k_l /(\rho _l c_l)$ is the temperature diffusivity, $\sigma _0$ is the selection constant, $\beta$ is the strength of surface energy anisotropy, and $\Sigma _{ds}$ is the solute concentration at the dendre surface. This concentration can be estimated as a mean solute concentration in the two-phase layer, i.e.

$$\begin{aligned} \Sigma _{ds} = \psi _*^{-1}\int \limits _0^{\psi _*}\Sigma (\psi ) d\psi . \end{aligned}$$

Thus, the average interdendritic spacing can be estimated using the analytical solutions of mushy layer equations with a weak incoming melt flow.

## Behaviour of solutions

**Figure 2:**
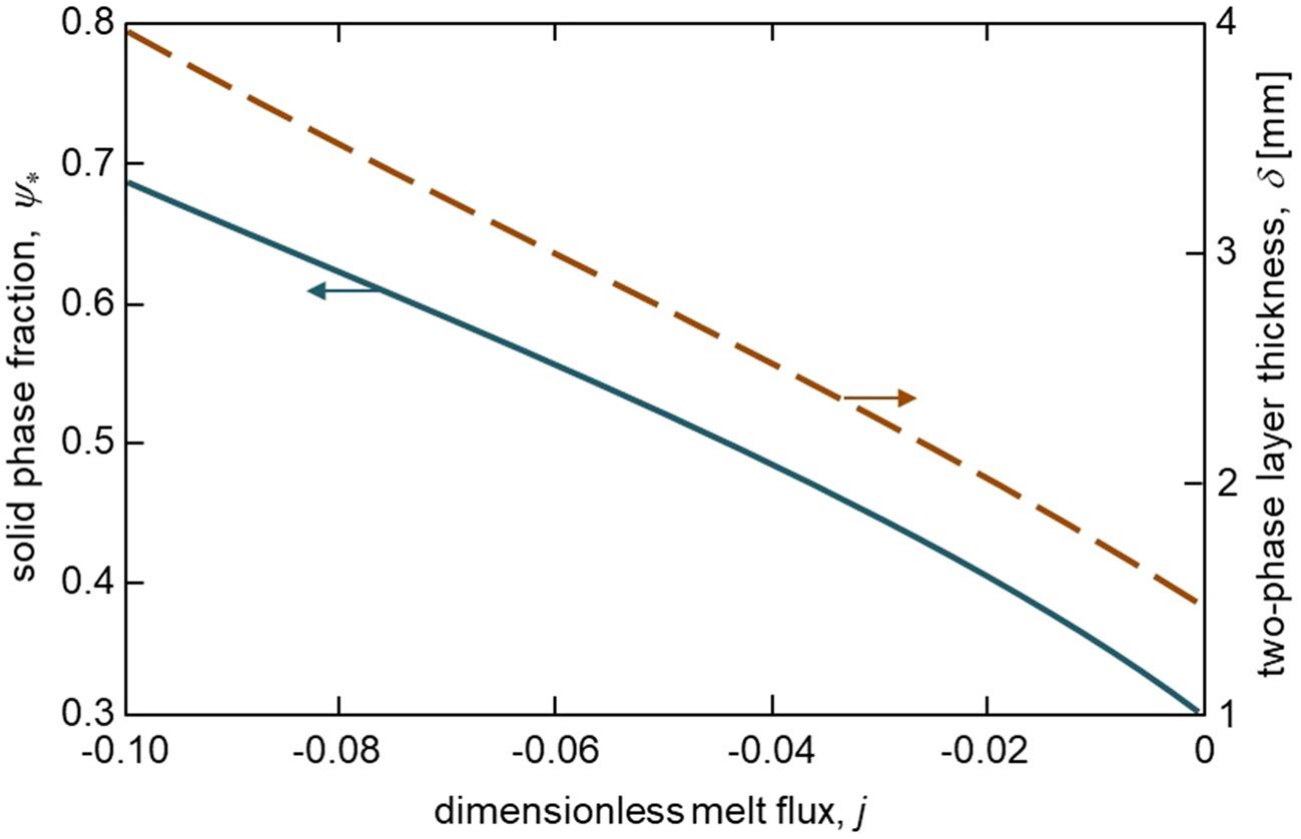
The solid phase fraction $\psi _*$ at the boundary $\xi =0$ and two-phase layer thickness $\delta$ versus dimensionless melt flux *j*. Physical parameters used for calculations correspond to the Fe-Ni melt^21^: $k_e=0.68$, $m_e=2.65$ K wt$\%^{-1}$, $Q_V =1.587\cdot 10^{10}$ J m$^{-3}$, $D_l=5\cdot 10^{-9}$ m$^2$ s$^{-1}$, $\rho _l =7\cdot 10^3$ kg m$^{-3}$, $\rho _s =7.8\cdot 10^3$ kg m$^{-3}$, $c_l =427.4$ J kg$^{-1}$ K$^{-1}$, $c_s =238.8$ J kg$^{-1}$ K$^{-1}$, $k_l=41.9$ J s$^{-1}$ K$^{-1}$ m$^{-1}$, $k_s=74.2$ J s$^{-1}$ K$^{-1}$ m$^{-1}$, $T_*=1803$ K, $C_\infty = 1$ wt$\%$, $g_s=400$ K m$^{-1}$.

**Figure 3:**
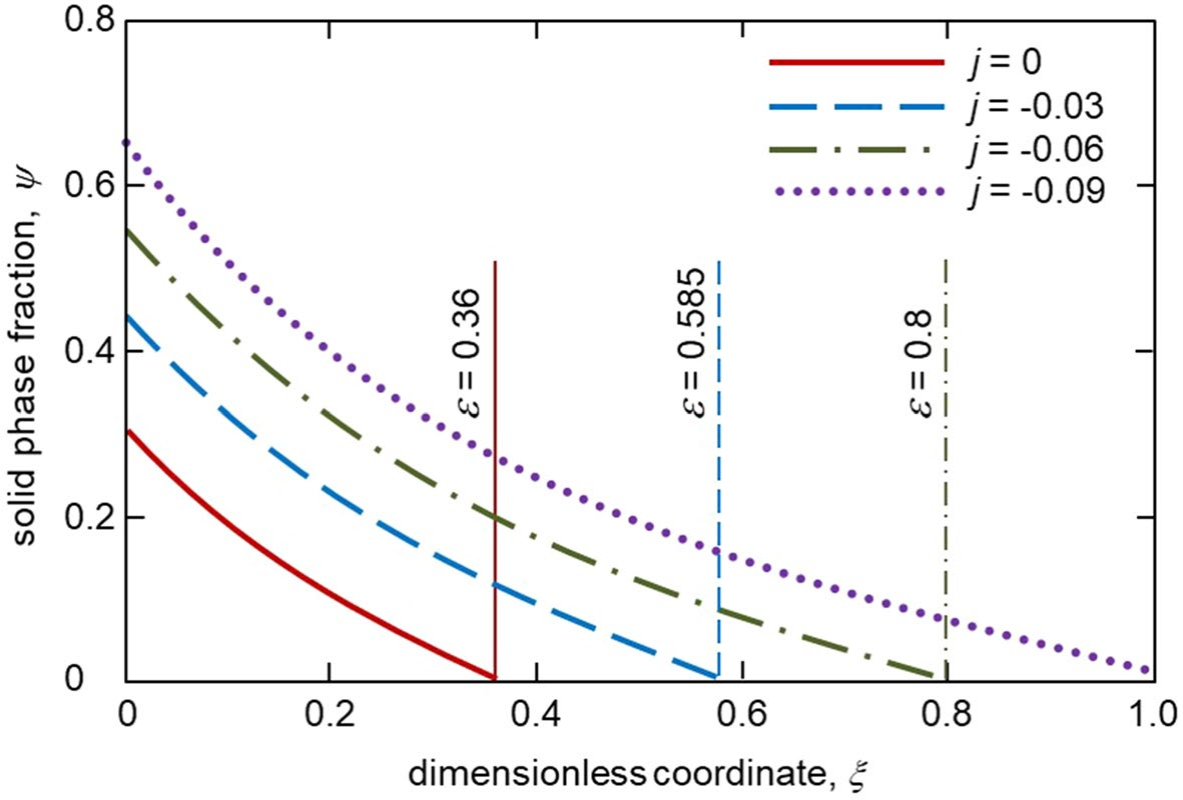
The solid phase fraction $\psi$ as a function of spatial coordinate $\xi$ in the two-phase layer for various melt fluxes *j*. Vertical lines show dimensionless thicknesses of the two-phase layer for various *j*.

**Figure 4:**
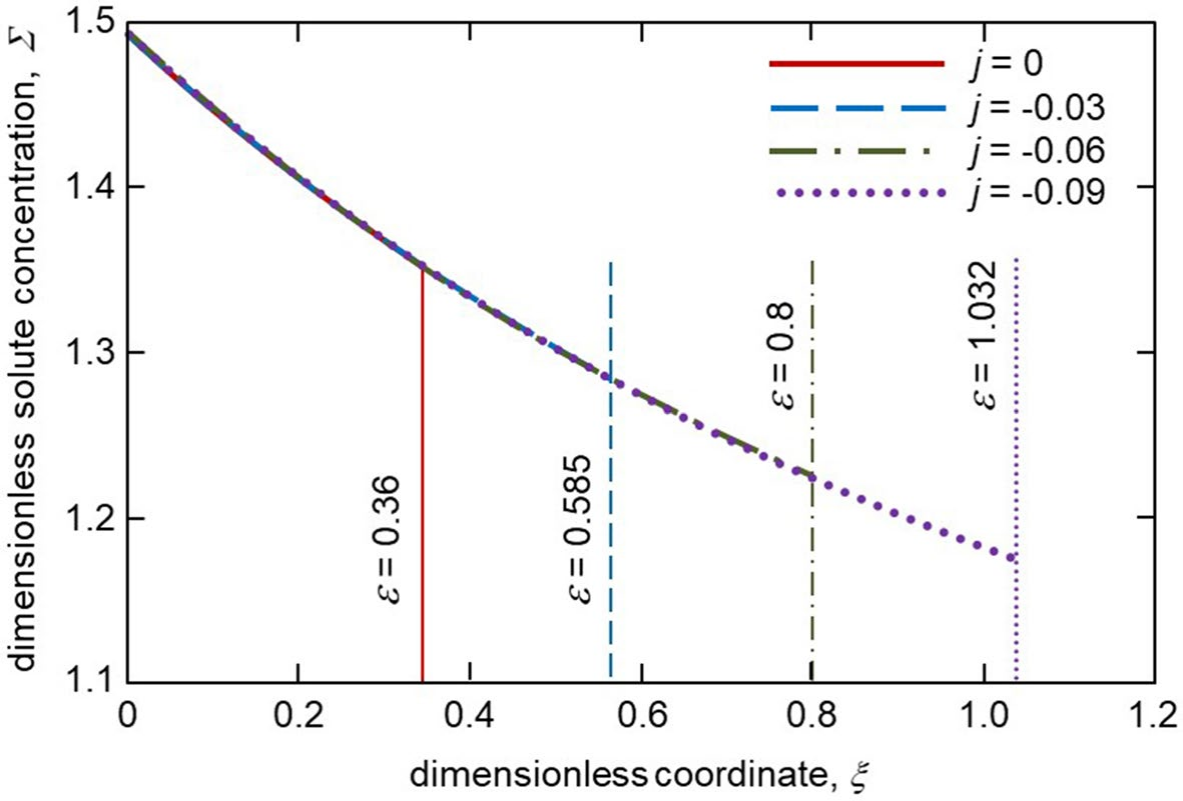
The solute concentration $\Sigma$ as a function of spatial coordinate $\xi$ in the two-phase layer for various melt fluxes *j*. Vertical lines show dimensionless thicknesses of the two-phase layer for various *j*.

Figures 2, 3 and 4 illustrate our analytical solution (15), (18), (19) and (22)–(29) for the undercooled Fe-Ni melt solidifying with a mushy layer. First of all, the solid phase fraction $\psi _*$ at the solid phase – two-phase layer interface is essentially dependent on the melt flux incoming to the two-phase layer. It can be seen that the greater the flux (higher the absolute value of *j*), the greater the boundary value of the solid phase fraction $\psi _*$. This is because a weak melt flow contributes to a more intense solidification of the melt and consequently increases the proportion of the solid phase in the two-phase layer. This is seen in Fig. 3 where the solid phase fraction profiles within the mushy layer are shown for various *j*. It is also easily seen that an increase in the solid phase fraction with increasing $\vert j\vert$ implies an increase in the two-phase layer thickness (see the vertical lines demonstrating the dimensionless two-phase layer thickness $\varepsilon$ in Fig. 3 and dashed line in Fig. 2 showing the dimensional thickness $\delta$).

**Figure 5:**
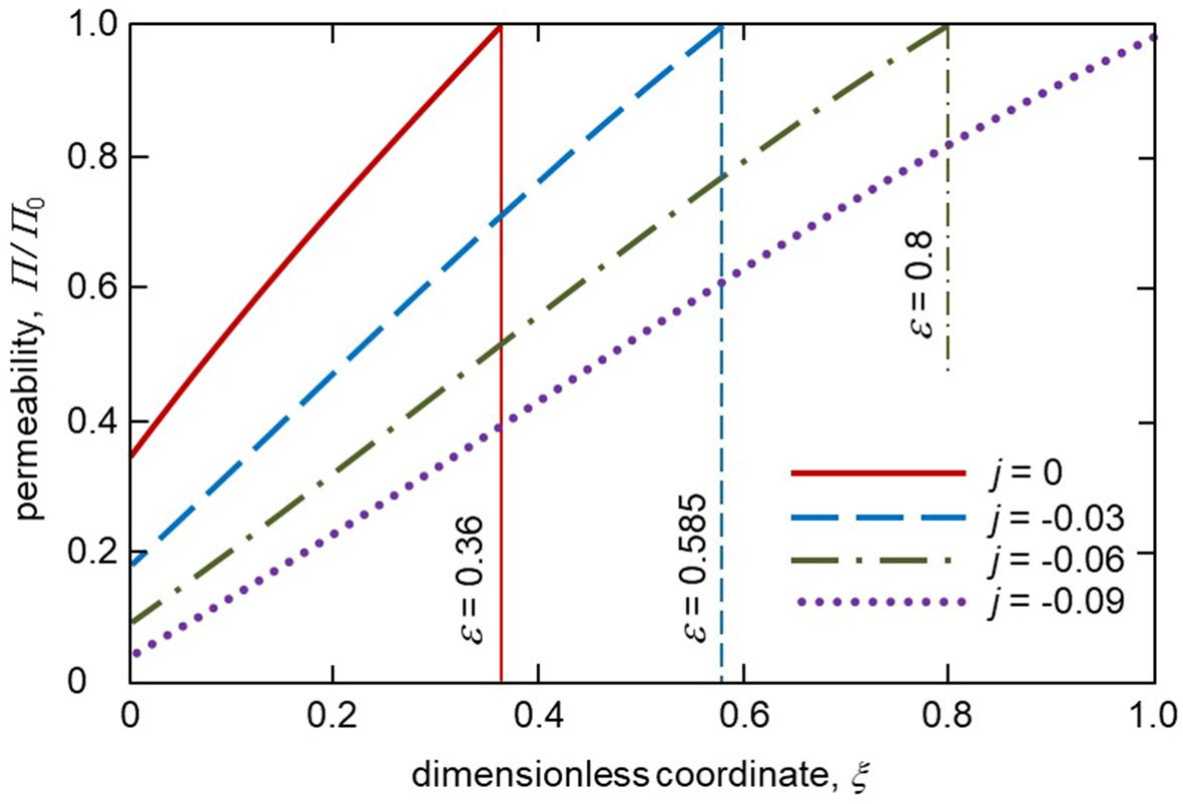
The two-phase layer permeability $\Pi /\Pi _0$ as a function of spatial coordinate $\xi$ for various melt fluxes *j*. Vertical lines show dimensionless thicknesses of the two-phase layer for various *j*.

Figure 4 shows the distribution of solute concentration in the mushy layer for different melt fluxes. As the thickness, $\varepsilon$ increases with increasing $\vert j\vert$ the solute concentration profile in a mush becomes wider. As this takes place, the boundary value of concentration at $\xi =\varepsilon$ decreases as $\vert j\vert$ increases. A greater extent of the two-phase layer means that the solid phase grows longer within this layer when the melt flux is higher. Figure 5 illustrates the mushy layer permeability plotted accordingly to expression (31). As is easily seen, the permeability becomes lower with increasing the melt flux $\vert j\vert$ due to an increase in the solid phase fraction within the mushy layer. The same behaviour is found for the average interdendritic spacing $\lambda _1$ described by expressions (32) and (33). Namely, $\lambda _1$ decreases with increasing $\vert j\vert$ (Table 1). This is because the mushy layer thickness becomes larger.

**Table 1:** Interdendritic spacing $\lambda _1$ at various melt fluxes *j* accordingly to expressions (32) and (33).

*λ*_1_·10^4^ [m]	8.756	8.18	7.678	7.208	6.737	6.251
*j*	0	− 0.02	− 0.04	− 0.06	− 0.08	− 0.1
$\psi _*$	0.302	0.4	0.482	0.554	0.622	0.686

Physical parameters are^16,33^: $d_a=1$, $d_0=7.8\cdot 10^{-10}$ m, $\sigma _0 =10$, and $\beta =0.66$.

## Conclusion

In summary, the problem of steady-state directional solidification with a two-phase layer is considered with allowance for a weak melt flow. To find analytical solutions, we assume that forced convection is one-dimensional and the process is established, i.e. nothing depends on time in the reference frame moving with a constant velocity together with a mushy layer. In the framework of a one-dimensional convective model under consideration, the flowing melt solidifies in the two-phase region and changes its internal structure. These model assumptions enable us to construct an analytical solution introducing a new independent variable - the solid phase fraction $\psi$. We show that the temperature and solute concentration as well as spatial coordinate in a mush are dependent only on $\psi$ in steady-state conditions. As this takes place, solidification velocity, two-phase layer thickness, permeability, and average interdendritic spacing are defined by the boundary value of solid fraction $\psi _*$. An important point is that the melt flow has a significant influence on all of these solutions. For example, the solid phase fraction, which decreases in the mushy layer from the solid phase boundary to the liquid phase boundary, increases as the flow rate $\vert j\vert$ grows. As a consequence, the mushy layer permeability and average interdendritic spacing decrease with increasing the melt flow. Physically it means that incoming melt solidifies more intensively in a mush with increasing $\vert j\vert$. This in turn leads to several times the greater thickness of the two-phase layer (phase transformation region).

The weak flow of the melt onto the two-phase layer leads to the formation of a new regime of directional crystallization and the analytical solution found here extends the theory of crystallization in a motionless melt^21^. Note that the one-dimensional convective theory developed takes place only at sufficiently low flow velocities of the melt, when $\vert j\vert \ll 1$ (when the melt flow velocity $\vert W \vert$ is much smaller than the crystallisation velocity *v*). As the melt velocity increases, the condition of complete solidification in a two-phase layer will be violated and two-dimensional hydrodynamic flow cells will form in the system^17,18^. A detailed study of this phenomenon requires investigation of the morphological stability of the two-phase layer equations taking into account the viscous fluid hydrodynamic equations and represents an important task for future research.

## Data Availability

All data generated or analysed during this study are included in this published article [and its supplementary information files].
